# Real-World Traffic-Polluted Air and Its Impact on a 3D Model of the Human Airway Epithelium

**DOI:** 10.3390/jox16030091

**Published:** 2026-05-22

**Authors:** Michal Sima, Helena Libalova, Zuzana Simova, Kristyna Vrbova, Antonin Ambroz, Jiri Klema, Lubos Dittrich, Michal Vojtisek-Lom, Pavel Rossner

**Affiliations:** 1 Department of Toxicology and Molecular Epidemiology, Institute of Experimental Medicine CAS, Videnska 1083, 142 00 Prague, Czech Republic; michal-sima@iem.cas.cz (M.S.); helena.libalova@iem.cas.cz (H.L.); zuzana.simova@iem.cas.cz (Z.S.); kristyna.vrbova@iem.cas.cz (K.V.); antonin.ambroz@iem.cas.cz (A.A.); 2 Department of Genetics and Microbiology, Faculty of Science, Charles University, Vinicna 5, 128 44 Prague, Czech Republic; 3 Department of Computer Science, Czech Technical University in Prague, Karlovo Namesti 13, 121 35 Prague, Czech Republic; klema@fel.cvut.cz; 4 Faculty of Mechatronics, Informatics and Interdisciplinary Studies, Technical University of Liberec, Studentska 1402/2, 461 17 Liberec, Czech Republic; lubos.dittrich@tul.cz (L.D.); michal.vojtisek@tul.cz (M.V.-L.)

**Keywords:** traffic, air pollution, lung tissue model, real-world exposure, cytotoxicity, transcriptomics

## Abstract

Exposure to air pollution is linked to adverse health outcomes. To better reflect real-world conditions, we employed a mobile exposure system enabling direct field exposure of the human airway epithelial model MucilAir™ to ambient air in a traffic-burdened locality. This study represents a follow-up to our previous work, in which a 5-day exposure period under extreme traffic-related pollution conditions resulted in premature cell loss. Under different meteorological conditions characterized by increased precipitation and lower particle number concentrations, MucilAir™ cultures were exposed to traffic-polluted air for 2 days. The exposure resulted in a mild but significant increase in cytotoxicity markers, including lactate dehydrogenase release and elevated levels of 15-F_2t_-isoprostane, indicating induction of the cellular stress response rather than severe cytotoxicity. A transcriptomic analysis revealed extensive gene expression changes; the enrichment of the pathways related to polycyclic aromatic hydrocarbon detoxification and amino acid biosynthesis suggests adaptive metabolic responses to oxidative and genotoxic stress. In parallel, the pathways associated with epithelial proliferation and repair, extracellular matrix organization, focal adhesion, and immune signaling were suppressed, indicating potential disruption of the epithelial homeostasis. Overall, these findings demonstrate that 2 days of exposure to real-world traffic-polluted air elicits adaptive stress responses in airway epithelial cells while simultaneously impairing the processes essential for epithelial integrity, potentially leading to airway dysfunction.

## 1. Introduction

Traffic is considered one of the main sources of air pollution in large urban areas, reflected by the substantial number of publications indexed in PubMed (over 7500 results for the term “traffic AND air pollution”) with a marked increase since 2000. Even though more environmentally friendly vehicle technologies have been introduced, the total number of vehicles on the roads continues to rise. The complexity of traffic-related pollution (e.g., gaseous components, particulate matter of various sizes, and associated chemical compounds, such as polycyclic aromatic hydrocarbons and heavy metals) makes in vitro research challenging. At the same time, such studies are urgently needed as exposure to particles smaller than 2.5 µm (PM2.5) has been associated with approximately 8 million premature deaths annually worldwide [1]. In addition to increased mortality, exposure to air pollution is linked to a wide range of adverse health outcomes. The International Agency for Research on Cancer has classified diesel and gasoline engine emissions as carcinogenic [2,3]. Chronic exposure to polluted air has been associated with an increased risk of neurodegenerative and cardiopulmonary diseases, while acute exposure exacerbates conditions such as asthma or chronic obstructive pulmonary disease [4].

Laboratory (in vitro) testing of traffic-polluted air remains challenging due to the complex and dynamic nature of emission mixtures. To overcome these limitations, advanced exposure approaches have been developed, particularly those based on air–liquid interface (ALI) systems that allow direct delivery of airborne pollutants to the apical surface of cells. Recent developments in ALI-based aerosol exposure systems have demonstrated controlled particle deposition and the induction of measurable biological responses, including cytotoxicity and gene expression changes in human airway epithelial cells [5]. The integration of ALI exposure with advanced 3D epithelial culture models further enhances physiological relevance and supports dose-dependent toxicological assessments of airborne particles [6]. In this context, our group developed a unique exposure chamber, which enabled in-house exposure of cells to complete engine exhaust emissions at air–liquid interface [7]. This system was subsequently applied to investigate the biological effects of gasoline exhaust emissions using both the advanced three-dimensional airway model MucilAir^TM^ and a conventional bronchial epithelial monolayer (BEAS-2B) cultured at ALI [8]. The results demonstrated differential responses between the models, with the MucilAir^TM^ system showing greater suitability for prolonged and physiologically relevant exposure conditions, allowing assessment of oxidative stress, epithelial barrier integrity, and gene expression changes. Consistent with these findings, MucilAir™ has been widely used in inhalation toxicology studies involving gaseous and aerosol exposures, providing detailed insight into cytotoxicity, inflammatory responses, and functional endpoints such as ciliary beating and epithelial integrity [9,10].

However, such systems are typically limited to laboratory-generated aerosols and do not fully reflect the complexity of real-world air pollution mixtures. The need for more advanced exposure approaches that integrate realistic environmental conditions, complex pollutant mixtures and advanced cellular models has been increasingly emphasized [11,12]. To address this gap, we recently modified the exposure system for direct field application, enabling controlled exposure of cell cultures to ambient air under real-world conditions [13,14]. Using this approach, multiple localities in the Czech Republic with distinct air pollution profiles (background, industrial, urban, and traffic) were investigated in repeated exposure campaigns employing MucilAir™ tissues derived from healthy and asthmatic donors. We demonstrated that real-world exposure conditions can induce measurable biological responses, including differences in immune response between healthy and diseased individuals [13]. The exposure platform was further utilized to assess the effects of industrial air pollution on primary olfactory mucosal cell cultures derived from patients with Alzheimer’s disease (AD) and healthy donors. In this model, exposure to industrial air pollution induced gene expression changes related to inflammatory responses in AD cells, indicating increased susceptibility of diseased tissue, whereas in healthy cells, the initiation of neurodegeneration-related processes was observed [14]. Collectively, these findings highlight the importance of integrating field-based exposure strategies with advanced human cell models to better understand the biological effects of complex air pollution mixtures.

Notably, as reported in a previous study, exposure of MucilAir™ tissues at a highly polluted traffic locality resulted in extensive cell death, likely due to elevated concentrations of ultrafine particles and nitrogen oxides. This severe cytotoxicity limited the ability to assess downstream molecular endpoints [13]. To overcome this limitation, we repeated the exposure experiment at the same traffic-burdened locality one year later using a modified experimental design. In the present study, we report the results of a shorter, 2-day exposure of MucilAir™ tissues derived from healthy donors, accompanied by detailed air-pollution measurements. We focused on cytotoxicity, epithelial integrity, oxidative stress-related responses, and transcriptomic alterations to further elucidate the mechanisms underlying toxicity induced by traffic-polluted air.

## 2. Materials and Methods

Due to the extensive cell death observed in the traffic locality after the 5-day exposure in our previous study [13], we repeated the experiment in this region under slightly modified conditions (the use of samples from healthy donors only and a shortened exposure period). The methods have been described in detail previously [13]; a brief description is provided below.

### 2.1. Cell Cultures, Exposure System, and Locality Description

The experiments were performed with the 3D model of human airway epithelium (MucilAir™; Epithelix Sàrl, Geneva, Switzerland) reconstituted from the primary human cells. In our study, inserts from five individual healthy, Caucasian, non-smoker donors (3 males, 2 females; considered as biological replicates) were used [some of them in more copies (based on the availability by the model producer at the time of the experiment) considered as technical replicates]. One male sample had to be excluded prior to exposure from the study due to cell death. Same set of inserts (in the same number of technical replicates) was utilized at the same time for exposure to ambient air (exposed) and for clean synthetic air (control). A description of the samples can be found in Table S1. The cell models were grown at the air–liquid interface at 37 °C, 5% CO_2_, and relative humidity > 90% in 24-well format Transwell^®^ cell culture inserts (Sigma-Aldrich, St. Louis, MO, USA) in culture medium provided by the cell model manufacturer. The culture medium was replaced every 2 days, and the cells were used one week after delivery.

The compact exposure chamber developed by our team was later modified for field experiments and successfully used in the previous study [13]. Small hermetically sealed boxes with standard 24-well plates, occupied by 7 cell inserts, are prepared in advance and brought to a portable incubator for exposure. There, two streams, one of outdoor air (exposed) and one of synthetic air (Linde plc, Dublin, Ireland; control), were enriched to 5% CO_2_, heated to 37 °C, humidified with membrane dryers (MD-700 series, Permapure, Lakewood, NJ, USA) and flown through the exposure boxes at 25 cm^3^/min per insert. All samples were used at the same time; therefore, identical exposure/control conditions were ensured for all inserts.

The exposure locality was identical and the experiment was performed at the same time of the year (mid-September; 17–18 September 2024) as described in [13]—Prague, the capital city of the Czech Republic, near a 6-lane road with an average number of 100,000 passing cars per day. Compared to the aforementioned study, the exposure (to ambient and control synthetic air) was shortened to a 2-day period, where each day, a 2 h exposure, 2 h resting period in the incubator, and another 2 h exposure took place (exposure from 6:45 to 8:45 AM and from 10:45 to 12:45 AM), which simulated a potential scenario of human exposure. Transepithelial electrical resistance (TEER) was measured prior to the exposure, and the cell medium was collected. The same procedure was applied at the end of the experiment, where the cells were also lysed. Between the first and second day, the cells were housed overnight, and in the morning of the second day, the cell medium was changed.

### 2.2. Online Air Pollution Analysis and Meteorological Conditions

The total number concentrations of particles were measured by a condensation particle counter (UF-CPC 200, Palas, Karlsruhe, Germany) with a 50% counting efficiency (d50) at 5 nm. A fast electric mobility particle sizer (Engine Exhaust Particle Sizer, EEPS, Shoreview, MN, USA) was used to measure particle size distributions in 32 channels over the 5.6–560 nm range at 1 Hz intervals.

The meteorological conditions, as a significant affecting factor, were retrieved from the CHMI 2024 yearbook (https://info.chmi.cz/rocenka/meteo2024/, accessed on 1 May 2026; in Czech). Precipitation, temperature and wind speed for September 2024 from Prague and the Central Bohemian Region were recorded.

### 2.3. Transepithelial Electrical Resistance, Cytotoxicity, and 15-F_2t_-Isoprostane Detection

Transepithelial electrical resistance (TEER) was measured by an EVOM2 ohm meter (World Precision Instruments, Sarasota, FL, USA) paired with an STX2 electrode (World Precision Instruments, Sarasota, FL, USA). The resistance values were calculated using the formula: TEER (ohm × cm^2^) = (resistance of the test tissue (ohm)-resistance value of the untreated membrane (ohm)) × surface area of the epithelium (cm^2^). The value of the untreated membrane was set at 100 ohm, and the epithelium surface area was 0.33 cm^2^. The measuring of TEER was performed before the experiment (T0) and after the final day of exposure (T2) for evaluation of the unaffected and affected cells.

Similarly, cytotoxicity (lactate dehydrogenase activity; LDH) by a Cytotoxicity Detection Kit (Roche, Basel, Switzerland) and the concentration of 15-F_2t_-isoprostane (IsoP) by the 8-isoprostane ELISA kit (Cayman Chemicals Company, Ann Arbor, MI, USA) were detected in the cell medium collected before (T0) and after (T2) the experiment. Both methods were performed in technical duplicate, and the absorbance was detected using SpetraMax^®^M5e (Molecular Devices, San Jose, CA, USA) set at 490 nm (LDH) or 405 nm (IsoP). The outcome of LDH was expressed as the absolute values of absorbance and, in the case of IsoP in pg, recalculated from the standard calibration curve with a detection range of 0.8–500 pg/mL. For LDH, the positive control (1% *v*/*v* Triton X-100, 1 h, and 37 °C) was used.

### 2.4. RNA Expression Analysis

After the exposure (T2), cells were lysed with RLT Buffer (Qiagen, Hilden, Germany), frozen in liquid nitrogen, and stored at −80 °C for RNA isolation. An AllPrep DNA/RNA/miRNA Universal Kit (Qiagen) was used for RNA extraction. A Fragment Analyzer System with RNA kit (both Agilent Technologies, Santa Clara, CA, USA) and a Qubit 4 fluorometer with Qubit RNA High Sensitivity Assay kit (both Thermo Fisher Scientific, Wilmington, DE, USA) were used for evaluating the RNA integrity and concentration, respectively.

The mRNA libraries were prepared by using 200 ng of the total RNA with the mRNA-seq Library Prep Kit for Illumina and the Poly(A) mRNA Capture module with 2xOligo d(T)25 capture Beads (both ABclonal Technology, Woburn, MA, USA). A Fragment Analyzer with an HS NGS Fragment Kit (Agilent Technologies, USA) and a Qubit 4 fluorometer with 1× dsDNA HS kit (Thermo Fisher Scientific) were utilized for library profile verification and concentration measurement, respectively. The sequencing of mRNA libraries was performed by a NextSeq™ 1000/2000 P2 XLEAP-SBS™ Reagent Kit (100 Cycles) on the NextSeq 1000/2000 system (all Illumina, Inc., San Diego, CA, USA).

### 2.5. Data and Statistical Analysis

An identical set of samples (Table S1) was used for exposure to ambient air and to clean synthetic air, which served as a control. Technical replicates derived from repeated donor samples were summed prior to analysis. The detected parameters (TEER, LDH, IsoP) were compared between T2 and T0 within the same group and between the exposed and control groups at the same time point using ANOVA, followed by Šídák’s multiple comparisons test.

mRNA sequencing data underwent processing via nf-core/rnaseq pipeline v3.18 [15] using the GRCh38 reference genome. The workflow incorporated FastQC (v0.12.1), TrimGalore! (v0.6.10), STAR (v2.7.11b), and Salmon (v1.11.0) for quality checks, trimming, alignment and quantification, respectively, with MultiQC (v1.29) used to compile quality metrics. Transcript- and gene-level expression matrices were generated via tximport. Differential expression analyses were performed in R version 4.6.0 (R Foundation for Statistical Computing, Vienna, Austria). DESeq2 (v1.51.6) [16] handled normalization and differential expression through median-of-ratios size-factor normalization, dispersion estimation with empirical Bayes shrinkage, negative binomial model fitting, Wald testing, and Benjamini–Hochberg correction. Technical replicates from repeated donors were summed before analysis. Gene filtering retained only those expressed in >3 samples, with the ≥1 sample having >50 reads. The analysis compared the exposed versus control groups using paired design with Donor.ID as the blocking factor. mRNA was considered as deregulated if it met these criteria: adjusted *p*-value < 0.05 and |log_2_FC| > 0.58.

Functional enrichment analyses and the construction of protein–protein interaction (PPI) networks of DEGs were performed using the Search Tool for the Retrieval of Interacting Genes/Proteins (STRING, v12.0) online database [17]. Information from multiple sources (e.g., literature, genome sequencing data, experimental databases, annotated biological pathways, and predicted co-expression datasets) is integrated into this tool to generate comprehensive interaction predictions. For a description of the link between two enzymes in the same metabolic pathway, medium confidence (an interaction score of 0.4) was set up.

## 3. Results

### 3.1. Particle Detection and Meteorological Conditions

**Figure 1:**
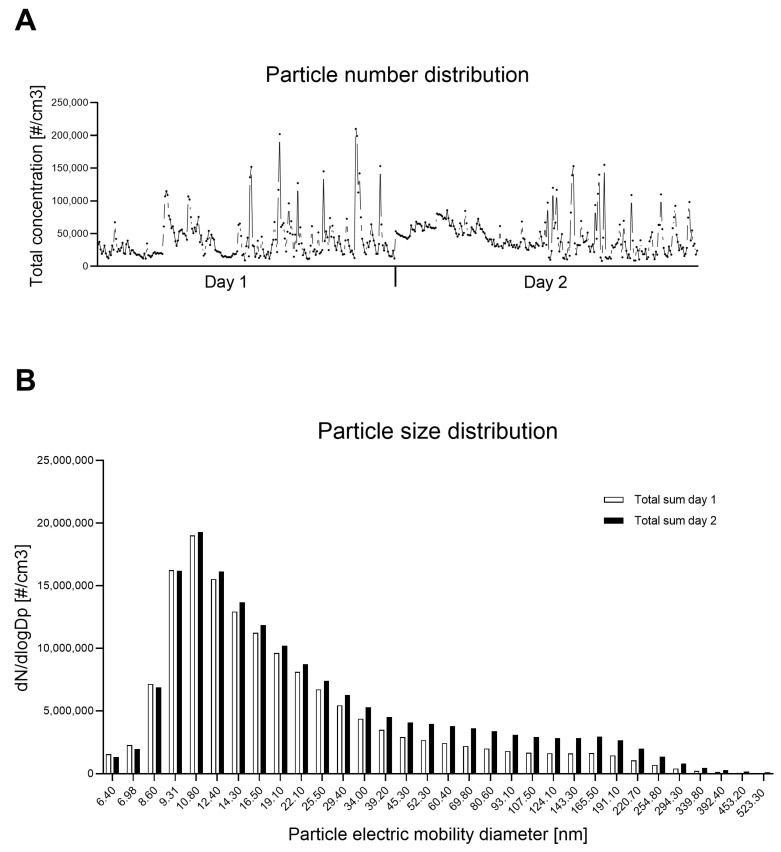
Particle number distribution (**A**) and particle size distribution (**B**) over the 2-day exposure period. Distributions were measured in 32 channels over the 5.6–560 nm range, and the total sums of particles are presented for each day.

The particle number concentration was measured during the whole exposure period. Several peaks reaching 2 × 10^5^ or 1.5 × 10^5^ #/cm^3^ were observed in the second half of the first and second day, respectively. One-minute averages of total concentrations did not usually exceed 1 × 10^5^ #/cm^3^ (Figure 1A). The particle size distribution patterns, plotted in Figure 1B, were similar on both exposure days, with a peak around 10 nm and majority of particles belonging to a nanoscale range (8–40 nm).

In September 2024, while the temperature and wind speed were close to normal values for this time of year, extremely high precipitations (almost 300% compared to normal) were recorded for this region (and for the Czech Republic—the highest comparison from 1961), which resulted in floods in several localities in the country (Table S2). These conditions differed from those of the previous year, when the weather in the traffic locality was mostly dry, with relatively high temperatures [13]. The average particle sums for each exposure period (5 days in 2023 and 2 days in 2024) are shown in Figure S1.

### 3.2. TEER, LDH, and IsoP

**Figure 2:**
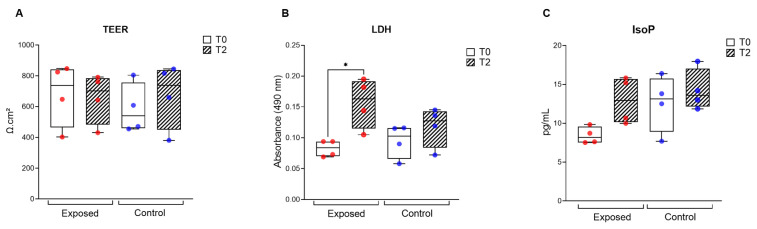
TEER (**A**), LDH (**B**), and IsoP (**C**) values in the exposed (red circles) and control (blue circles) samples before (T0) and after (T2) the exposure. Asterisks indicate significant differences (* *p* < 0.05).

TEER, LDH, and IsoP were measured before (T0) and after the exposure (T2) directly in cell inserts (TEER) or in the collected medium (LDH, IsoP). No significant difference in TEER level was observed, suggesting no integrity loss or tight junction damage. A mild increase in LDH and IsoP values was detected when the exposed samples were compared between T2 and T0 (Figure 2), indicating slight cytotoxicity and oxidative damage caused by the 2-day ambient air exposure.

### 3.3. Deregulation of Gene Expression

The expression of mRNA was compared between the samples exposed for two days to ambient air (exposed) and the controls maintained in synthetic air (controls). Differentially expressed genes (DEGs) were identified according to the criteria described in Materials and Methods (Section 2.5).

The 2-day exposure to traffic-polluted air resulted in the deregulation of 81 mRNAs, of which 56 were downregulated and 25 upregulated (Table S3). The three most strongly downregulated mRNAs were CD300A (CD300a molecule), NDUFA4L2 (NDUFA4 mitochondrial complex-associated like 2), and PGF (Placental growth factor). Alternatively, MKI67 (Marker of proliferation Ki-67), ENSG00000257767 (Novel protein), and NOS2 (Nitric oxide synthase 2) showed the strongest upregulation.

**Figure 3:**
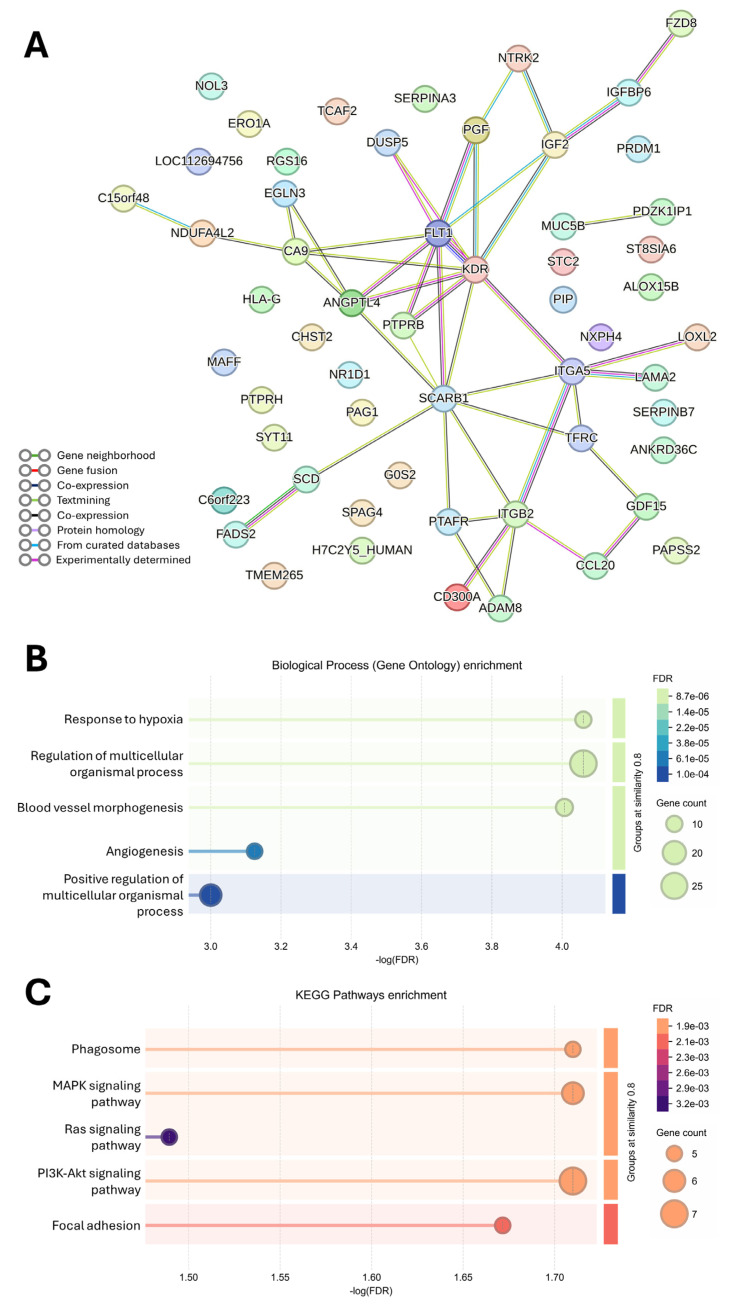
PPI network analysis of downregulated DEGs. PPI network created by STRING (**A**). The top 5 GO Biological processes (**B**) and KEGG pathways (**C**) are shown. Each term is ranked according to its significance, expressed as—log (FDR), and grouped based on the similarity threshold of 0.8.

The STRING analysis of 56 downregulated DEGs revealed the formation of one larger protein–protein interaction network (PPI) comprising 27 DEGs and one smaller PPI network comprising two DEGs. The remaining 27 DEGs were not interconnected (Figure 3A). Reduced expression of these DEGs contributed to the inhibition of 66 GO processes, from which the most significant were “Response to hypoxia”, “Regulation of multicellular organismal process”, and “Blood vessel morphogenesis”. Similarly, six KEGG pathways were inhibited, with the top three including “MAPK signaling pathway”, “Phagosome”, and “PI3K-Akt signaling pathway” (Figure 3B,C; a complete list is shown in Table S4).

**Figure 4:**
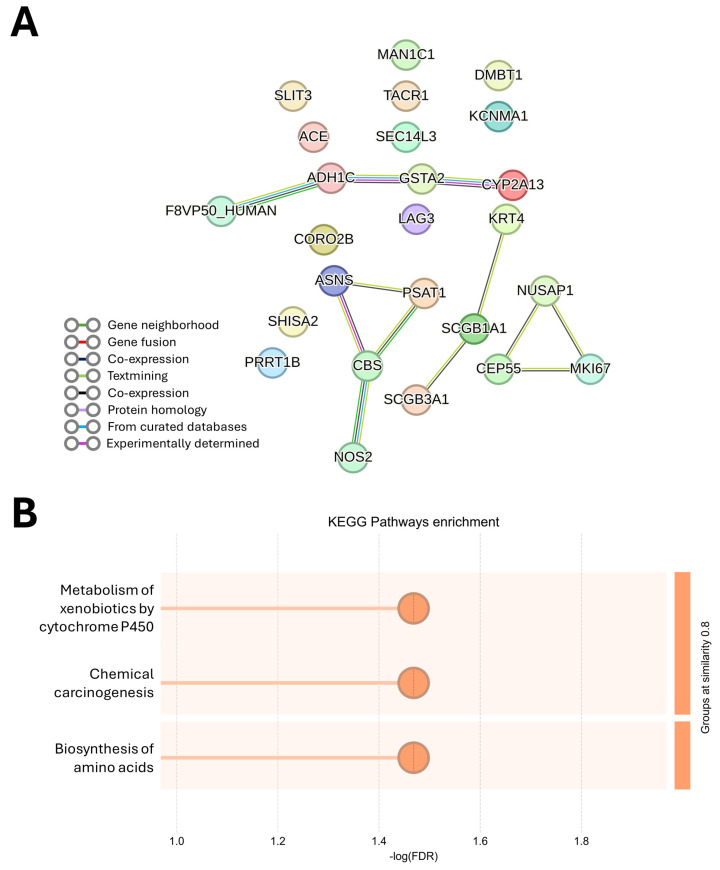
PPI network analysis of upregulated DEGs. PPI network created by STRING (**A**) and the top three KEGG pathways (**B**) are indicated. Each term is ranked according to its significance, expressed as-log (FDR) and grouped based on similarity threshold of 0.8.

The same analysis was performed for the 25 upregulated DEGs. Four small networks (2 composed of 4 DEGs and 2 composed of 3 DEGs) were constructed, while 11 DEGs remained unconnected (Figure 4A). No significantly enriched GO biological processes were discovered, but three KEGG pathways (Metabolism of xenobiotics by cytochrome P450, Biosynthesis of amino acids and Chemical carcinogenesis) were induced by the exposure (Figure 4B, an overview is shown in Table S4).

## 4. Discussion

To date, many studies investigating the toxicity of airborne particles rely on traditional in vitro designs that use acute, high-dose exposures and simplified cellular models. These approaches are useful for hazard identification; however, they may overestimate acute toxicity and miss the biologically relevant chronic and low-dose effects associated with real-world exposure. Recently, we employed a unique mobile exposure system that enables direct field exposure of MucilAir™ cells to real-world ambient air. Using this approach, we have previously investigated the biological effects of ambient air in four distinct localities with differing pollution profiles (industrial, urban, traffic, and background) following a 5-day exposure period [13]. In the traffic locality, air pollution from vehicle emissions prevailed and due to meteorological conditions characterized by hot and sunny weather without precipitation, exceptionally high total particle number concentrations—also in comparison with other localities—and NOx concentrations more than two-fold above the exposure limits were detected. These conditions most likely led to the premature loss of cells that were intended to be exposed for 5 days. In the current follow-up study, a different meteorological situation (increased precipitation, lower total particle number concentrations), lower traffic (due to a road closure) and a shorter exposure interval (2 days) allowed us to identify the mechanisms underlying the biological response of the MucilAir™ model to air pollution in the traffic locality.

Toxicity assays, including TEER, LDH and IsoP, serve as cellular markers of integrity and health in MucilAir™ cultures. A mild but significant increase in cytotoxicity and IsoP levels may indicate a cellular stress response and adaptive mechanism rather than overt damage. To uncover more molecular mechanisms underlying this stress, transcriptomic analysis was performed.

We observed induction of KEGG pathways “Metabolism of PAHs” and “Chemical carcinogenesis” with contributing genes *CYP2A13, GSTA2* and *ADH1C*. Air pollution exposure is known to contribute to carcinogenesis, largely mediated by PAHs and their nitro-derivatives. Cytochrome P450—particularly members of the CYP1 family, and, to a lesser extent, also CYP2A13—participate in the metabolic activation of PAHs, generating reactive intermediates and reactive oxygen species (ROS) that induce DNA, lipid, and protein damage. In contrast, glutathione S-transferases (GST), including GSTA2, catalyze the conjugation of PAH metabolites with glutathione and thus facilitate their detoxification. PAH dihydrodiol metabolites can also be processed by alcohol dehydrogenases such as ADH1C to form catechols, which contribute to oxidative damage through redox cycling [18]. Upregulated *PSAT1, ASNS,* and *CBS* encode key enzymes in “Biosynthesis of amino acid”, another overrepresented KEGG pathway. PSAT1 is central to serine biosynthesis, linking glycolytic carbon metabolism to redox balance, while ASNS produces asparagine, supporting cellular survival under nutrient or metabolic stress. CBS regulates sulfur amino acid metabolism and homocysteine levels and is functionally linked to the folate-dependent pathways involved in antioxidant defense [19]. Together, the induction of PAH metabolism and amino acid biosynthesis pathways suggest enhanced detoxification, antioxidant defense and metabolic adaptation in response to environmental stress.

The pathway enrichment analysis also indicated inhibition of the “Phagosome” pathway, driven by decreased expression of genes associated with epithelial particle uptake, vesicular trafficking, and immune interaction (*TFRC*, *ITGA5*, *SCARB1*, *ITGB2*, *HLA-G*). Although airway epithelial cells are not professional phagocytes, these genes contribute to receptor-mediated endocytosis, scavenger receptor activity, cell–matrix interactions and epithelial immunomodulation. Their coordinated downregulation possibly suggests an adaptive cellular mechanism that limits particle internalization and inflammation. However, it may also, in turn, contribute to epithelial barrier dysfunction, airway remodeling or altered immune regulation [20,21]. Similarly, inhibition of “Focal adhesion” pathway-related genes, including *LAMA2*, *KDR*, *ITGA5*, *PGF*, and *FLT1*, indicates disrupted cell–matrix interactions and impaired focal adhesion signaling that may result in pathological conditions such as fibrosis, cancers, skin, and autoimmune disorders [22]. Downregulation of growth factor ligands and receptors, including *KDR, FLT1, NTRK2, IGF2,* and *PGF,* collectively contributed to the reduced activation of KEGG pathways “MAPK signaling pathways”, “PI3K–Akt signaling pathways” and “Ras signaling pathways”, suggesting suppressed proliferative and repair signaling in epithelial cells under stress.

We also observed downregulation of multiple genes involved in GO processes “Angiogenesis”, “Hypoxia response” and “Blood vessel morphogenesis”, including *KDR, ITGA5, PGF, FLT1, ANGPTL4, PTPRB, LOXL2,* and *EGLN3*. These genes encode VEGF receptors, integrins and growth factors representing central regulators of angiogenesis, vascular morphogenesis, and endothelial responses to hypoxia [23]. Since MucilAir™ lacks endothelial cells, these findings possibly reflect the suppression of hypoxia-responsive signaling, extracellular matrix remodeling, and growth factor pathways rather than angiogenesis. Such epithelial dysfunctions may disrupt barrier integrity, repair processes, and epithelial–immune crosstalk, with important implications for airway pathology. Air pollution exposure has been linked to changes in mucociliary activity, barrier function, airway inflammation, epithelial–mesenchymal transitions, and airway remodeling [24].

Finally, the enrichment of GO processes “Regulation of multicellular organismal process” and “Positive regulation of multicellular organismal process” likely reflects altered epithelial signaling programs that normally regulate multicellular organization and tissue homeostasis.

## 5. Study Limitations and Future Perspectives

This study has several limitations that should be acknowledged.

(1) The number of donor-derived samples was limited by the capacity of the exposure box used for field-based exposure experiments. The study included tissues derived from four donors (two males and two females), with some samples represented by technical replicates due to experimental constraints. Although the limited number of donors did not allow robust analysis of sex-specific or donor-specific effects, the use of primary donor-derived tissues increases the biological relevance of the model by partially reflecting natural human variability. Importantly, despite the relatively low number of biological replicates, statistically significant and biologically consistent changes in gene expression profiles were observed between the exposure conditions. However, the reduced sample size may limit statistical power and increase uncertainty in effect size estimates; therefore, these findings should be interpreted with appropriate caution, while still suggesting that the experimental setup was capable of capturing biologically relevant molecular responses to traffic-related air pollution. In addition, a high degree of experimental standardization and reproducibility is difficult to achieve in field-based exposure studies, as real-world ambient air composition is strongly influenced by changing meteorological conditions, traffic intensity, and seasonal variability. The use of primary donor-derived tissues further contributes to natural biological variability between experiments. Nevertheless, these aspects better reflect the complexity of real-life human exposure conditions.

(2) The exposure duration in the traffic locality was shortened to preserve cell viability after extensive cytotoxicity that had been observed during longer exposures in our previous study. Consequently, the presented data primarily reflect early biological responses to traffic-related air pollution and may not fully capture effects associated with prolonged or chronic exposure scenarios. 

(3) The study predominantly relied on transcriptomic analyses, while additional protein validation or functional assays were constrained by the small amount of biological material obtained from differentiated primary ALI cultures and by technical challenges associated with handling exposed tissues following field experiments. Under these conditions, transcriptomic profiling represented a highly sensitive and robust approach for detecting subtle molecular alterations induced by real-world air pollution exposure. Future studies should include larger donor cohorts, extended exposure periods, and more detailed chemical characterization of the exposure atmosphere to better identify the specific drivers of the observed molecular responses. Integration of transcriptomic analyses with proteomic and functional endpoints would further improve understanding of the mechanisms underlying air pollution-induced toxicity.

## 6. Conclusions

The present study represents a follow-up to our previous work in which 5-day exposure to traffic-related air pollutants was lethal for MucilAir™ cells. Under the modified exposure conditions applied in this study, a mild increase in toxicity markers (LDH, IsoP) was observed, indicating a cellular stress response but not extensive cellular damage. We further revealed widespread changes in gene expression in MucilAir™ after the 2-day exposure to traffic-polluted air, reflecting increased detoxification of PAHs and amino acid biosynthesis in response to oxidative and genotoxic stress, which may facilitate short-term adaptation. However, the suppression of pathways linked to proliferation and repair signaling, extracellular matrix organization, focal adhesion, and immune/inflammatory signaling could, in turn, contribute to long-term epithelial dysfunctions. Overall, these findings reveal that airway epithelial cells exhibit short-term adaptive mechanisms in response to real-world traffic-polluted air, but also potential vulnerability to impaired epithelial integrity and function. Importantly, despite several limitations, our study demonstrates the suitability of the MucilAir™ model combined with the mobile exposure system for mechanistic investigations under physiologically relevant, field-based conditions.

## Data Availability

The original contributions presented in this study are included in the article/Supplementary Material. Further inquiries can be directed to the corresponding author.

## Supplementary Materials

The following supporting information can be downloaded at: https://www.mdpi.com/article/10.3390/jox16030091/s1, Table S1: A detailed description of the analyzed samples; Table S2: An overview of the meteorological conditions for Prague and the Central Bohemian Region (regarding wind for the Czech Republic) in September 2024; Table S3: A list of differentially expressed mRNAs. The green and red colors indicate down- and upregulation, respectively; Table S4: An overview of the enrichment analysis of deregulated genes. The alteration of GO biological processes and KEGG pathways for downregulated DEGs as well as KEGG pathways for upregulated DEGs is indicated; Figure S1: A comparison of particle average size distribution over the current 2-day (2024) and previous 5-day (2023) exposure periods in the traffic locality.

## References

[1] Pozzer A., Anenberg S.C., Dey S., Haines A., Lelieveld J., Chowdhury S. (2023). Mortality Attributable to Ambient Air Pollution: A Review of Global Estimates. GeoHealth.

[2] (2014). Diesel and Gasoline Engine Exhausts and Some Nitroarenes. IARC Monographs on the Evaluation of Carcinogenic Risks to Humans.

[3] Turner M.C., Godderis L., Guénel P., Hopf N., Quintanilla-Vega B., Soares-Lima S.C., Chaiklieng S., Da Silva J., Fustinoni S., Gi M. (2025). Carcinogenicity of Automotive Gasoline and Some Oxygenated Gasoline Additives. Lancet Oncol..

[4] Zanobetti A., Ryan P.H., Coull B.A., Luttmann-Gibson H., Datta S., Blossom J., Brokamp C., Lothrop N., Miller R.L., Beamer P.I. (2024). Early-Life Exposure to Air Pollution and Childhood Asthma Cumulative Incidence in the ECHO CREW Consortium. JAMA Netw. Open.

[5] Buckley A., Guo C., Laycock A., Cui X., Belinga-Desaunay-Nault M.-F., Valsami-Jones E., Leonard M., Smith R. (2024). Aerosol Exposure at Air-Liquid-Interface (AE-ALI) in Vitro Toxicity System Characterisation: Particle Deposition and the Importance of Air Control Responses. Toxicol. Vitr..

[6] Küstner M.J., Eckstein D., Brauer D., Mai P., Hampl J., Weise F., Schuhmann B., Hause G., Glahn F., Foth H. (2024). Modular Air–Liquid Interface Aerosol Exposure System (MALIES) to Study Toxicity of Nanoparticle Aerosols in 3D-Cultured A549 Cells in Vitro. Arch. Toxicol..

[7] Vojtisek-Lom M., Dittrich L., Pechout M., Cervena T., Vimrova A., Sikorova J., Zavodna T., Ondracek J., Aakko-Saksa P., Topinka J. (2025). Portable emissions toxicity system: Evaluating the toxicity of emissions or polluted air by exposure of cell cultures at air-liquid interface in a compact field-deployable setup. Sci. Total Environ..

[8] Rossner P., Cervena T., Vojtisek-Lom M., Vrbova K., Ambroz A., Novakova Z., Elzeinova F., Margaryan H., Beranek V., Pechout M. (2019). The Biological Effects of Complete Gasoline Engine Emissions Exposure in a 3D Human Airway Model (MucilAir(TM)) and in Human Bronchial Epithelial Cells (BEAS-2B). Int. J. Mol. Sci..

[9] Sharma M., Stucki A.O., Verstraelen S., Stedeford T.J., Jacobs A., Maes F., Poelmans D., Van Laer J., Remy S., Frijns E. (2023). Human Cell-Based *in Vitro* Systems to Assess Respiratory Toxicity: A Case Study Using Silanes. Toxicol. Sci..

[10] Czekala L., Wieczorek R., Simms L., Yu F., Budde J., Trelles Sticken E., Rudd K., Verron T., Brinster O., Stevenson M. (2021). Multi-Endpoint Analysis of Human 3D Airway Epithelium Following Repeated Exposure to Whole Electronic Vapor Product Aerosol or Cigarette Smoke. Curr. Res. Toxicol..

[11] Upadhyay S., Palmberg L. (2018). Air-Liquid Interface: Relevant In Vitro Models for Investigating Air Pollutant-Induced Pulmonary Toxicity. Toxicol. Sci..

[12] Lacroix G., Koch W., Ritter D., Gutleb A.C., Larsen S.T., Loret T., Zanetti F., Constant S., Chortarea S., Rothen-Rutishauser B. (2018). Air–Liquid Interface *In Vitro* Models for Respiratory Toxicology Research: Consensus Workshop and Recommendations. Appl. Vitr. Toxicol..

[13] Rossner P., Libalova H., Cervena T., Sima M., Simova Z., Vrbova K., Ambroz A., Novakova Z., Elzeinova F., Vimrova A. (2025). Real-World Outdoor Air Exposure Effects in a Model of the Human Airway Epithelium—A Comparison of Healthy and Asthmatic Individuals Using a Mobile Laboratory Setting. Ecotoxicol. Environ. Saf..

[14] Rossner P., Libalova H., Sima M., Cervena T., Simova Z., Vrbova K., Ambroz A., Rossnerova A., Novakova Z., Elzeinova F. (2026). Molecular Alterations in Human Olfactory Mucosal Cells from Healthy Individuals and Individuals with Alzheimer’s Disease Induced by Real-World Ambient Air. Environ. Toxicol. Pharmacol..

[15] Ewels P.A., Peltzer A., Fillinger S., Patel H., Alneberg J., Wilm A., Garcia M.U., Di Tommaso P., Nahnsen S. (2020). The Nf-Core Framework for Community-Curated Bioinformatics Pipelines. Nat. Biotechnol..

[16] Love M.I., Huber W., Anders S. (2014). Moderated Estimation of Fold Change and Dispersion for RNA-Seq Data with DESeq2. Genome Biol..

[17] Szklarczyk D., Kirsch R., Koutrouli M., Nastou K., Mehryary F., Hachilif R., Gable A.L., Fang T., Doncheva N.T., Pyysalo S. (2023). The STRING Database in 2023: Protein–Protein Association Networks and Functional Enrichment Analyses for Any Sequenced Genome of Interest. Nucleic Acids Res..

[18] Moorthy B., Chu C., Carlin D.J. (2015). Polycyclic Aromatic Hydrocarbons: From Metabolism to Lung Cancer. Toxicol. Sci..

[19] Do L.K., Lee H.M., Ha Y.-S., Lee C.-H., Kim J. (2025). Amino Acids in Cancer: Understanding Metabolic Plasticity and Divergence for Better Therapeutic Approaches. Cell Rep..

[20] Yuan L., Liu H., Du X., Yao Y., Qin L., Xia Z., Zhou K., Wu X., Yuan Y., Qing B. (2023). Airway Epithelial ITGB4 Deficiency Induces Airway Remodeling in a Mouse Model. J. Allergy Clin. Immunol..

[21] Zaborek-Łyczba M., Łyczba J., Mertowska P., Mertowski S., Hymos A., Podgajna M., Niedźwiedzka-Rystwej P., Grywalska E. (2021). The HLA-G Immune Checkpoint Plays a Pivotal Role in the Regulation of Immune Response in Autoimmune Diseases. Int. J. Mol. Sci..

[22] Kanno S., Oda N., Abe M., Terai Y., Ito M., Shitara K., Tabayashi K., Shibuya M., Sato Y. (2000). Roles of Two VEGF Receptors, Flt-1 and KDR, in the Signal Transduction of VEGF Effects in Human Vascular Endothelial Cells. Oncogene.

[23] Carmeliet P., Jain R.K. (2011). Molecular Mechanisms and Clinical Applications of Angiogenesis. Nature.

[24] Kayalar Ö., Rajabi H., Konyalilar N., Mortazavi D., Aksoy G.T., Wang J., Bayram H. (2024). Impact of Particulate Air Pollution on Airway Injury and Epithelial Plasticity; Underlying Mechanisms. Front. Immunol..

