# Supplementary figures and images for: Real-World Traffic-Polluted Air and Its Impact on a 3D Model of the Human Airway Epithelium
Source: J Xenobiot. 2026 May 22;16(3):91. doi: 10.3390/jox16030091 (PMC13214726; doi:10.3390/jox16030091)

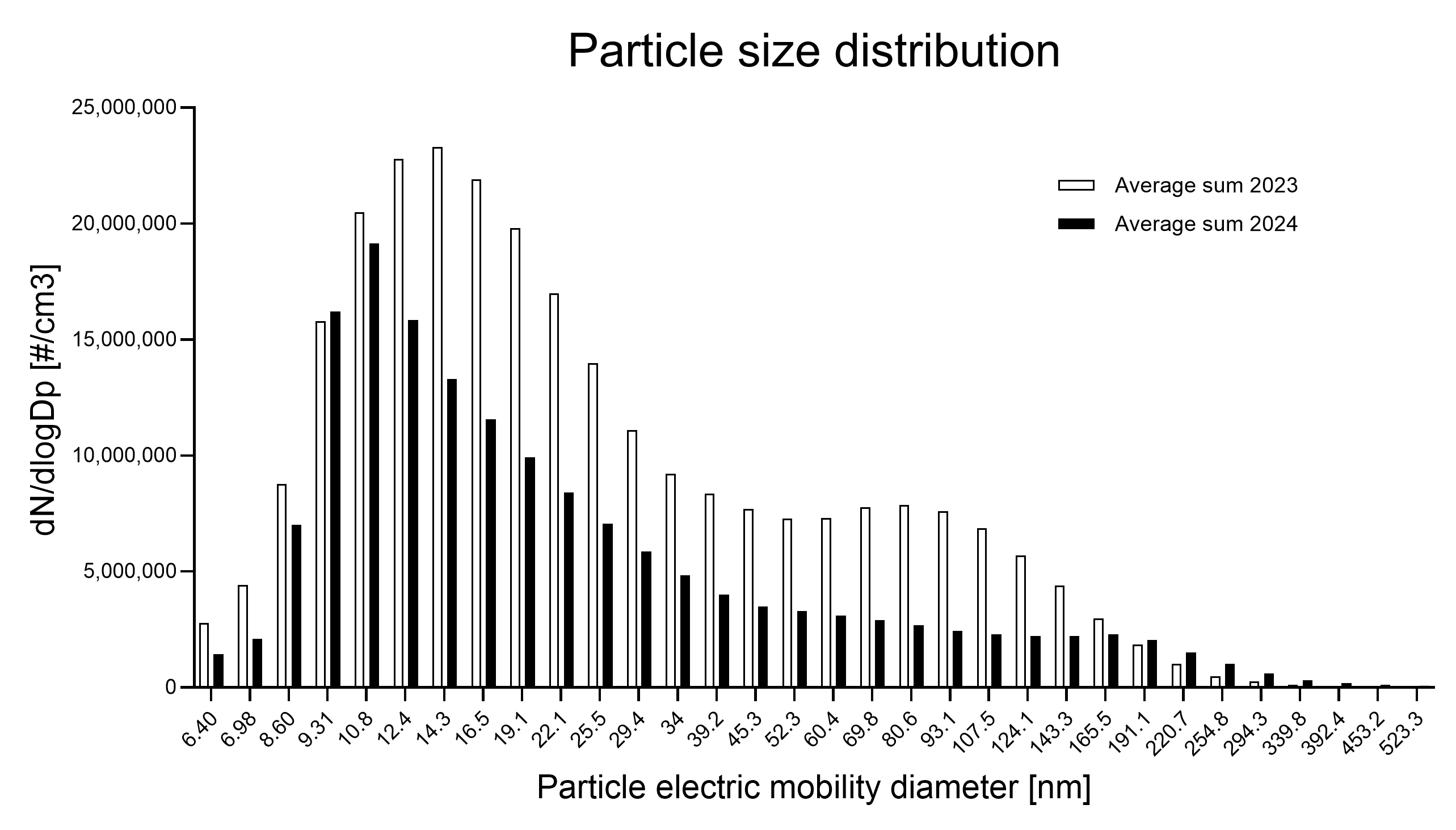

Supplement: Supplementary file 1 [file jox-16-00091-s001.zip › Figure S1.png]
